# Utility of Microscopic Investigations

**Published:** 1845-09

**Authors:** 


*Utility of Microscopic Investigations.*—Dr. Hughes Bennett, while treating of the importance of histology in diagnosis, observes that not long ago a man was admitted into the Royal Infirmary at Edinburgh, with complete paralysis of one side of the body, and partial paralysis on the other. He died, and on examining the brain to determine the nature of the lesion, nothing whatever could be seen, but on considering the symptoms, and the suddenness of the paralysis of both sides at once, Dr. H. Bennett was induced to re-examine the structure of the pons Varolii and medulla oblongata, and found in the former undoubted evidence of the existence of inflamed softening, combined with numerous exudation corpuscles. In another case, one of abscess in the arm, its approaching resolution was diagnosed from the appearance of fibrinous granules in the urine. M. Vogel, of Munich, in two cases, one of malignant disease, and the other of supposed malignant disease, availed himself of the aid of the microscope in forming his diagnosis with great advantage. In the first case, an ulcerated tumor near the angle of the jaw, the discharge, on examination, was found to consist, first of an amorphous matter, with blood corpuscles, and drops of fat; secondly, of crystals of cholesterine; and thirdly, of numerous cells, about one-fiftieth of a millimeter in diameter, containing a large nucleus and a nucleolus. They were not flat, but globular. The character of these cells led him to pronounce the tumor malignant, an opinion which its increasing growth, the death of the indvidual, and a *post-mortem* examination confirmed. The other case was that of a woman, fifty years of age, of cachectic appearance, who had an ulcerated breast of six months standing. The ulcer was about an inch from the nipple, sunk deep into the substance of the organ, and about the size of a walnut. Its edges and the surrounding substance were firm and indurated. The glands in the axilla were slightly enlarged; the other breast healthy. It being requisite to ascertain the real character of this ulcer, before proceeding to an operation, an examination of the fluid upon the surface of the ulcer was made under the microscope, and exhibited, first, pus-cells, which on the addition of acetic acid, presented the usual granular nucleus. Secondly, there were several flux scales, presenting all the characters of pavement epithelium. Thirdly, there were cells of an elongated form, similar to those observed in granulations and cellular tissue in an early stage. From these circumstances it was diagnosed that the ulcer was not malignant, and it subsequently disappeared under the use of common applications.—*Ibid.*

